# Adipose Stem Cells Display Higher Regenerative Capacities and More Adaptable Electro-Kinetic Properties Compared to Bone Marrow-Derived Mesenchymal Stromal Cells

**DOI:** 10.1038/srep37801

**Published:** 2016-11-24

**Authors:** Ahmed El-Badawy, Marwa Amer, Reda Abdelbaset, Sameh N. Sherif, Marwan Abo-Elela, Yehya H. Ghallab, Hamdy Abdelhamid, Yehea Ismail, Nagwa El-Badri

**Affiliations:** 1 Center of Excellence for Stem Cells and Regenerative Medicine (CESC), Zewail City of Science and Technology, Egypt; 2 Center of Nanoelectronics and Devices (CND), Zewail City of Science and Technology/American University in Cairo, Cairo, Egypt; 3 Department of Biomedical Engineering, Helwan University, Cairo, Egypt

## Abstract

Adipose stem cells (ASCs) have recently emerged as a more viable source for clinical applications, compared to bone-marrow mesenchymal stromal cells (BM-MSCs) because of their abundance and easy access. In this study we evaluated the regenerative potency of ASCs compared to BM-MSCs. Furthermore, we compared the dielectric and electro-kinetic properties of both types of cells using a novel Dielectrophoresis (DEP) microfluidic platform based on a printed circuit board (PCB) technology. Our data show that ASCs were more effective than BM-MSCs in promoting neovascularization in an animal model of hind-limb ischemia. When compared to BM-MSCs, ASCs displayed higher resistance to hypoxia-induced apoptosis, and to oxidative stress-induced senescence, and showed more potent proangiogenic activity. mRNA expression analysis showed that ASCs had a higher expression of Oct4 and VEGF than BM-MSCs. Furthermore, ASCs showed a remarkably higher telomerase activity. Analysis of the electro-kinetic properties showed that ASCs displayed different traveling wave velocity and rotational speed compared to BM-MSCs. Interestingly, ASCs seem to develop an adaptive response when exposed to repeated electric field stimulation. These data provide new insights into the physiology of ASCs, and evidence to their potential superior potency compared to marrow MSCs as a source of stem cells.

Mesenchymal stromal cells (MSCs) hold great potential in regenerative medicine based on their self-renewal properties and multi-lineage differentiation capacity^1^. MSCs have been isolated from various sources such as bone marrow, adipose tissue, umbilical cord, umbilical cord blood and other adult tissues^2^. However, bone marrow (BM) MSCs, and recently, adipose stem cells (ASCs) are the most suitable cells in clinical trials because of their easy access and lack of ethical concerns. Several studies reported similar morphological characteristics and cell surface markers for both BM-MSCs and ASCs, but significant biological differences with regards to their proliferation rate and differentiation capacities^3^,^4^,^5^,^6^,^7^. Moreover, significant differences between BM-MSCs and ASCs in their cytokine secretome and chemokine expression have been observed^8^,^9^,^10^. Despite the few reports that compared the biology of BM-MSCs and ASCs^9^,^11^,^12^,^13^, no comparison to evaluate the difference in electrical properties between both type of cells was reported.

While bone marrow mononuclear^14^,^15^,^16^,^17^,^18^ cells and endothelial progenitor cells^19^,^20^ have been applied with promising results in cardiovascular diseases, MSCs appear to be more efficient for the treatment of limb ischemia^21^. MSCs have the capacity to differentiate into cell types including osteoblasts, chondrocytes, adipocytes, endothelial cells, and vascular smooth muscle cells, but their destiny is largely determined by the local microenvironment^22^. In addition to multipotency, MSCs secrete several proangiogenic growth factors, especially in a microenvironment of low oxygen concentration^23^. Several *in vitro* studies^24^,^25^,^26^ and *in vivo* studies^27^,^28^,^29^,^30^ show that potency of MSCs in vasculogenesis, particularly during ischemia, as hypoxia induces MSCs to form capillary-like structures *in vitro*^23^. However, ongoing clinical trials on the use of stem cells to treat ischemic conditions lack standardization. In this study, we compared the therapeutic potential and electrical differences of MSCs derived from both bone marrow and adipose tissue. *In vitro* studies aim to determine biological characteristics of both cells that may contribute to their *in vivo* function.

## Results

### Therapeutic potential of BM-MSCs and ASCs in a rat model of hind-limb ischemia

**Figure 1:**
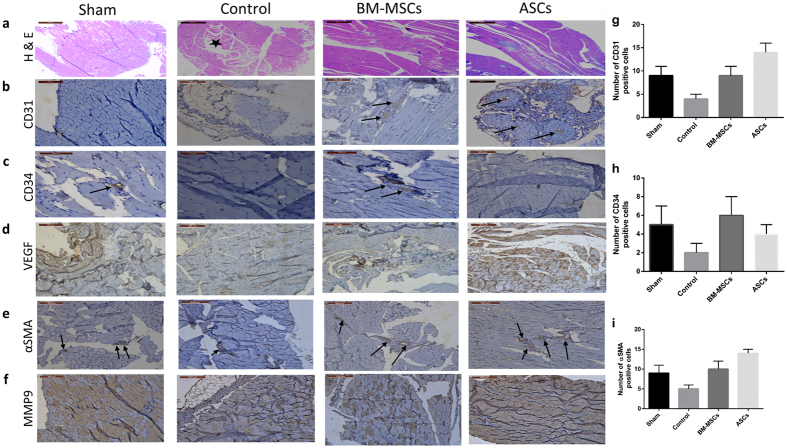
Representative histological analysis of hind limb muscles: Gastrocnemius muscles were collected after 4 weeks of cell therapy. Tissue samples were stained with: (**a**) H & E showing muscle degeneration in the ischemic control group and infiltration of lymphocytes (*) compared to normal looking muscles in the BM-MSCs and ASCs treated groups (**b**) Positive staining for-CD31, in transplanted mice, especially In the ASCs-transplanted group **(c)** CD34 expression is pronounced in the BM-MSC-transplanted group (**d**) Increased expression of VEGF especially in the ASC-treated group **(e)** Staining with anti-αSMA is more pronounced in the ASCs group (**f**) staining of both tissues with anti-MMP9. Quantitative evaluation of the expression levels of CD31 (**g**), CD34 (**h**) and αSMA (**i**) was evaluated by counting the number of positive cells in each group. Data are shown as mean ± S.D. (error bars). Scale bars, 200 μm.

BM-MSCs and ASCs were characterized by their cell surface marker expression using flow cytometry and by their adipogenic and osteogenic differentiation potential (Supplemental Fig. 1B & C). Both BM-MSCs and ASCs were shown to be positive for CD29, CD90 and were negative to CD45 surface antigens (Supplemental Fig. 1D). This expression profile is in accordance with the International Society for Cellular Therapy Statement of minimal criteria for defining MSC^31^. To compare the *in vivo* differences between BM-MSCs and ASCs in promoting angiogenesis in an animal model of hind limb ischemia, the gastrocnemius muscles were collected 3 weeks after administration of either ASCs, or BM-MSCs. H & E staining showed muscle degeneration and lymphocyte infiltration in the ischemic control group while muscles in limbs treated with both BM-MSCs as well as ASCs were protected after cell transplantation (Fig. 1a). Immunohistological staining for CD31 and CD34 antigens showed increase of the number cells expressing these antigens (endothelial cells and endothelial progenitor cells respectively) in the ASC-treated group and the BM-MSC-treated group, respectively. (Fig. 1b and c). On the other hand, VEGF expression was especially prominent in the ASC-treated group (Fig. 1d). Immunostaining for αSMA, a marker of vascular smooth muscle cells, and MMP9, which is essential for neovascularization and initiating angiogenesis was higher in the ASC-transplanted group (Fig. 1e and f). The expression of CD31, CD34 and αSMA was quantified by counting the number of positive cells (Fig. 1g, h and i). Representative histological analysis of original and magnified images of hind limb muscles stained for CD31, CD34, VEGF, αSMA and MMP9 are shown in Supplemental Figures 2–6.

### ASCs are more resistant to oxidative stress-induced senescence than BM-MSCs

**Figure 2:**
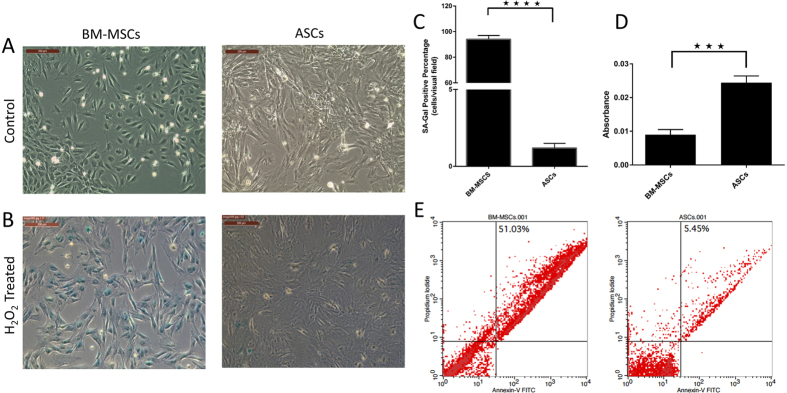
ASCs are more resistant to oxidative stress-induced senescence than BM-MSCs: BM-MSCs and ASCs were exposed to oxidative stress by treating cells with a dose of 600 μM H_2_O_2_. (**A**) Control cells, (**B**) H_2_O_2_ treated cells showing more than 90% of BM-MSCs positive for SA-β-gal, and ASCs negative for SA-β-gal. (**C**) Representative images are displayed and data are shown as mean ± S.D. (error bars) of counted SA-Gal positive cells from 5 microscopic fields of 4 independent replicates. *****p < 0.0001. Scale bars, 200 μm. (**D**) MTT assay (5 mg/ml) to evaluate the proliferation rate of BM-MSCs and ASCs after H_2_O_2_ treatment. Formazan absorbance at 570 nm with reference to 630 nm expressed as a measure of cell proliferation [***p < 0.05]. (**E**) Cellular apoptosis after H_2_O_2_ treatment was measured by FACS analysis using Annexin-V and PI staining.

Oxidative stress has been reported to induce cellular aging and decline in organ function. Hydrogen peroxide (H_2_O_2_) is widely used to induce oxidative stress-induced premature senescence in which cells undergo a change in morphology, acquire larger, more flattened shape, and express the senescence-associated β-galactosidase (SA-β-gal)^32^. However, as a small portion of cells can recover from a single H_2_O_2_ stress and re-enter cell cycle^33^, BM-MSCs and ASCs were exposed to a second dose of H_2_O_2_. After H_2_O_2_ treatment, although no changes in cell morphology were observed, more than 90% of BM-MSCs became positive for SA-β-gal indicating the onset of cellular senescence (Fig. 2A and B), while ASCs were partially protected from oxidative stress-induced senescence as determined by a mostly negative staining for SA-β-gal (Fig. 2C). To examine the effect of oxidative stress on BM-MSCs and ASCs proliferation, we used an MTT assay. After exposure to H_2_O_2_, ASCs maintained a significantly higher proliferation rate compared to BM-MSCs (Fig. 2D, p < 0.05). Annexin-V and PI staining showed that the cellular death after H_2_O_2_ treatment was 51.03% in BM-MSCs while was only 5.45% in ASCs (Fig. 2E), suggesting that ASCs may be partially protected from oxidative stress-induced senescence.

### ASCs are more resistant to hypoxia-induced apoptosis compared to BM-MSCs

**Figure 3:**
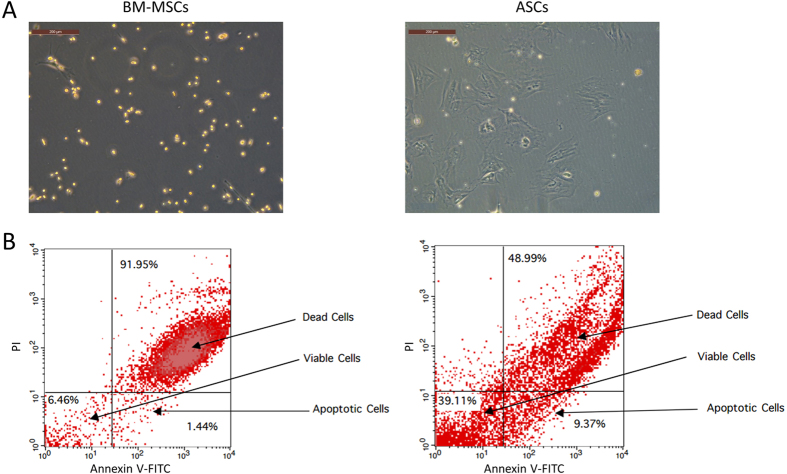
ASCs are more resistant to *in vitro* hypoxia apoptosis compared to BM-MSCs: BM-MSCs and ASCs were exposed to 1% O_2_ for 24 hours and then measured for morphological changes and apoptotic resistance. (**A**) BM-MSCs showed significant changes in cell morphology as cells became pyknotic while ASCs maintained their elongated, spindle fibroblast-like phenotype. (**B**) Cellular apoptosis after hypoxia conditioning was measured by FACS analysis using Annexin-V and PI staining (*n* = 3).

During ischemia, deprivation of oxygen is an important contributor to cellular death. To investigate the effect of severe hypoxia on BM-MSCs and ASCs, we exposed both types of cells to 1% O_2_ (hypoxia). Our results show that 24 hours after hypoxia, significant changes in cell morphology of BM-MSCs were observed as cells became pyknotic, while ASCs maintained their elongated, spindle fibroblast-like shape (Fig. 3A). Cellular apoptosis after hypoxia was measured by Annexin-V and PI staining which showed that cellular death was 91.95% in the BM-MSCs while only 48.99% in ASCs (Fig. 3B). Moreover, there was a significant difference in the percentage of apoptotic cells between BM-MSCs and ASCs (1.44% and 9.37% respectively). These data suggest that ASCs have a superior tolerance to hypoxia-induced cytotoxicity than BM-MSCs.

### ASCs have a more potent proangiogenic activity compared to BM-MSCs

**Figure 4:**
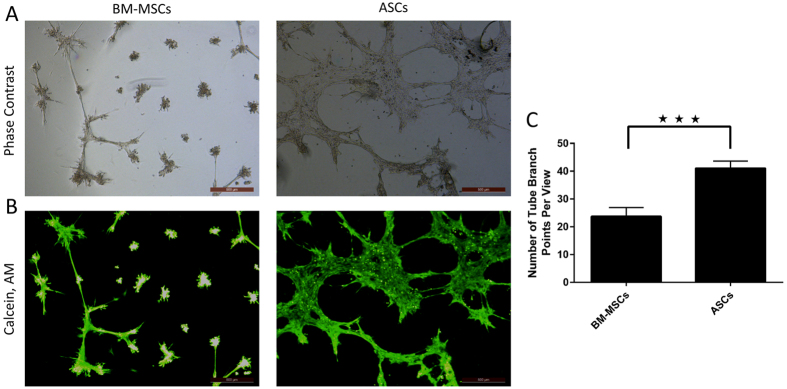
ASCs have a more potent pro-angiogenic effects when compared to BM-MSCs). Upper panel is the phase contrast image of the field shown on the below panel (A). Tubular structures were photographed (**A and B**) and then quantified by counting the number of branch points (**C**). *In vitro* tube formation assay (**A and B**) and tube branch points (**C**). Representative images are displayed and data are shown as mean ± S.D. (error bars) of counted tube branch points from 4 microscopic fields of three independent replicates. ***p < 0.005. Scale bars, 500 μm.

The angiogenic capacities of both BM-MSCs and ASCs were assessed using an *in vitro* angiogenesis tube formation after culture on Geltrex-coated plates in large vessel endothelial-supplemented Medium 200. As shown in Fig. 4A, within 12 hours, ASCs formed more extensive networks of hallow, capillary tube-like structures, compared to BM-MSCs. The number of branching points was significantly higher in ASCs than BM-MSCs (p < 0.005, Fig. 4B).

### ASCs have a high activity of telomerase compared to BM-MSCs

**Figure 5:**
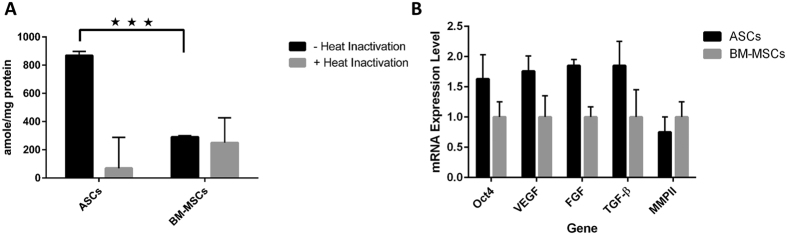
(**A**) ASCs have a significantly higher telomerase activity than BM-MSCs: Quantification of telomerase activity in BM-MSCs and ASCs was assayed using a fluorescent PCR-based telomerase activity detection method, TRAP (Telomeric Repeat Amplification Protocol). Telomerase activity is shown as amole of product/mg protein. (**B**) mRNA expression levels in ASCs with reference to BM-MSCs: qPCR analysis showed a higher expression of Oct4, VEGF, FGF, and TGF-β in ASCs while MMPII expression was higher in BM-MSCs. Data are shown as mean ± S.D. (error bars). ****p < 0.005.

We assayed telomerase activity in cell lysates of BM-MSCs and ASCs using a PCR-based telomerase activity detection method, TRAP (Telomeric Repeat Amplification Protocol). Figure 5A shows significantly higher telomerase activity in ASCs compared to BM-MSCs (*p* < 0.005).

### mRNA expression using quantitative real-time PCR

Expression of mRNAs for stem cell and angiogenic markers Oct3/4, VEGF, TGF-β, FGF2 and MMPII in BM-MSCs and ASCs was examined by qRT-PCR (Fig. 5B). Expression of Oct3/4, TGF-β and FGF2 was significantly higher in ASCs compared to BM-MSCs (p < 0.01). However, MMPII expression was higher in BM-MSCs than in ASCs while VEGF expression in ASCs was 2-fold higher than in BM-MSCs (p < 0.01).

### Traveling Wave Speed of ASCs is higher than BM-MSCs

A microfluidic platform was used to determine two electrokinetic features of ASCs and BM-MSCs: traveling wave speed and rotation speed. 100 μL of each type of cells were pipetted on the top of the electrode array. The microfluidic chamber was placed on top to seal the electrode array. 90-degree shifted signals were fed to the travelling wave electrodes. The motion of each type of cells was monitored through the microscope camera system and simultaneously recorded while the frequency of the applied signals was scanned from 1 KHz to 8 MHz.

**Figure 6:**
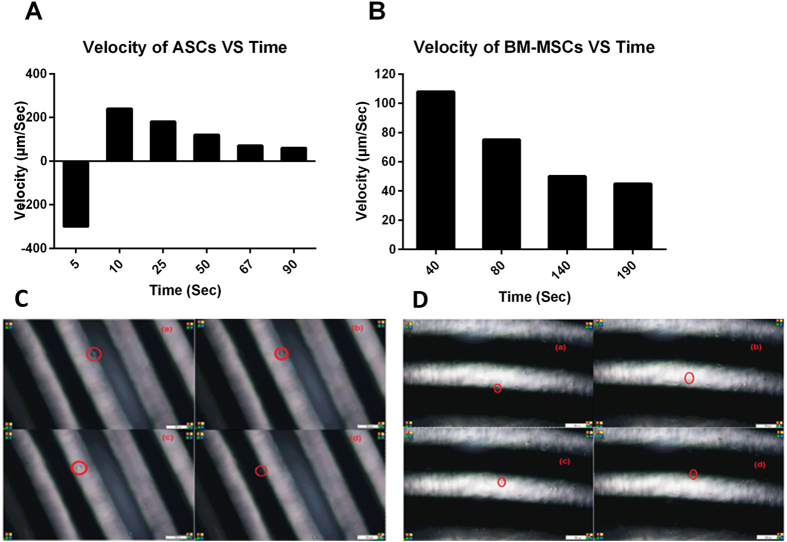
Traveling wave velocity of (**A**) ASCs and (**B**) BM-MSCs at 8 MHz during different time points: Snap shots of (**C**) ASCs and (**D**) BM-MSCs at frequency 8 MHz, 10 Vpp square wave.

The traveling wave speed was measured for both ASCs and BM-MSCs at frequency of 8 MHz. The speed for each type of cells versus time was measured and plotted (Fig. 6A and B). ASCs displayed a higher starting velocity than BM-MSCs, while the rate of decrease for ASCs was faster than that for BM-MSCs (Fig. 6A and B). Supplementary Videos 1 & 2 show the travelling velocity of ASCs and BM-MSCs, respectively.

On energizing the electrodes, the following behaviors were observed:

- ASCs traveled first in an opposite direction (i.e., against the planned and designed direction) and then changed its direction towards the designed direction.
- BM-MSCs always traveled in the planned direction.
- The response time (i.e., the time consumed by the cells to start moving after turning on the electric field) for BM-MSCs was shorter than that for ASCs.

### ASCs have better adaptation to electrical fields than BM-MSCs

In another test, the electric field was turned on and off. After a few seconds, the electric field was turned on again. Interestingly, upon the second electric stimulation, ASCs did not move in any direction, while BM-MSCs showed response in the form of movement in the same original direction of movement. This suggests that ASCs may have developed an adaptation mechanism to the applied electrical field. Figure 6C and D show snapshots for ASCs and BM-MSCs, respectively, at different locations of the electrode array, where the traveling wave speed could be obtained.

**Figure 7:**
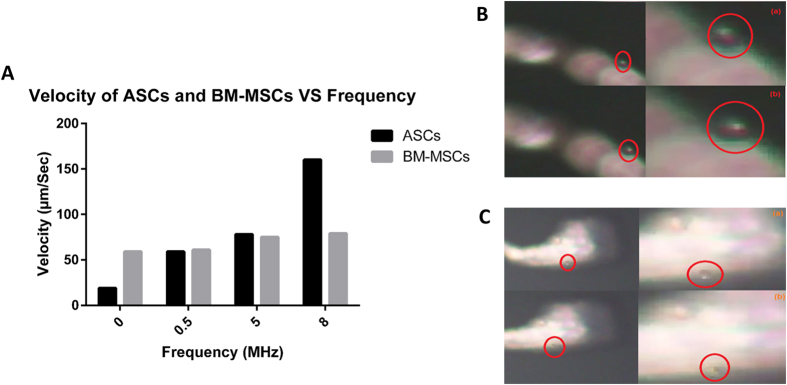
(**A**) Velocity of ASCs and BM-MSCs versus frequency. Snap shots of rotation of (**B**) ASCs and (**C**) BM-MSCs at 8 MHz, 10 Vpp.

A constant amplitude of electric current was then applied (10 V peak to peak) to both types of cells while changing the frequency of the applied electric field. ASCs displayed slower velocity at low frequency compared to BM-MSCs. However, ASCs exhibited higher speed at higher frequencies, compared to BM-MSCs (Fig. 7A).

### Rotation Speed of ASCs is lower than BM-MSCs

The rotation speed for cells were checked under the same electric field conditions (i.e., 10 V peak to peak and 8 MHz frequency). The measured rotation speed for ASCs and BM-MSCs was 305.1°/sec and 1764.71°/sec, respectively (Fig. 7B and C).

## Discussion

BM-MSCs are widely used in cell therapies because of their abundance, easy propagation, and lack of ethical concerns in their applications. Recently, adipose tissue has emerged as a viable source for stem cells since it contains a significantly higher frequency of ASCs, and tissue collection is simple and accessible. In this study, we compared BM-MSCs and ASCs in an effort to determine a potentially better candidate for cell therapy in regenerative medicine applications, especially ischemic conditions.

*In vitro*, both ASCs and BM-MSCs showed similar morphological features, adipogenic and osteogenic differentiation potential and cell surface marker expression. *In vivo*, while both cells protected the ischemic muscles from degeneration and lymphocyte infiltration, immuno-histological staining showed differences. Staining for CD31 (an endothelial cell marker), VEGF (a potent angiogenic factor), SMA (a marker of vascular smooth muscle cells) and MMP9; which is essential for neovascularization and initiating angiogenesis, was more pronounced in tissues transplanted with ASCs. These data indicate that ASCs may present a more potent source for promoting neovascularization in ischemia tissue. It is noteworthy however that expression of CD34; a marker for endothelial progenitor cells, was more pronounced in the BM-MSC transplanted group. The underlying mechanisms responsible for the therapeutic superiority of ASCs in ischemia remain unknown. Recent studies have shown that the angiogenic potential of ASCs and BM-MSCs is mediated by several factors, including differentiation into vascular endothelial cells, and recruitment other progenitor cells, and production of various cytokines and angiogenic factors, including VEGF and bFGF^34^,^35^,^36^.

The ability of stem cells to resist senescence and apoptosis is especially important where cells are transplanted to a damaged area for therapeutic purposes. In this study, BM-MSCs and ASCs were exposed to superoxide oxidative stress, which induces cellular aging and *in vitro* hypoxia to emulate *in vivo* ischemic conditions. ASCs showed superior resistance to oxidative stress-induced senescence and hypoxia-induced apoptosis compared to BM-MSCs. However, the mechanisms involved in these differences in stress-induced injury are not fully understood.

Recent studies have demonstrated that the angiogenic potential of ASCs and BM-MSCs is related to their ability to produce various angiogenic factors, including VEGF and bFGF^30^,^37^. In this study, ASCs showed more robust tube formation in basement membrane matrix indicating a better differentiation potential into vascular endothelial cells. This was confirmed by quantitative real-time PCR analysis of angiogenesis and pluripotency related genes, which showed ASCs to have a higher expression of Oct-4, VEGF, FGF2 and TGF-β compared to BM-MSCs.

Unlike most normal somatic cells, that are telomerase negative, low to moderate levels of the enzyme have been described in adult stem cells from skin^38^ gut^39^ and from the hematopoietic system^40^,^41^. Furthermore, telomerase activity in hematopoietic cells has been associated with a capacity for self-renewal^42^. In this study we compared the telomerase activity of BM-MSCs and ASCs from the same passage, and found that ASCs have a significantly higher telomerase activity than BM-MSCs, however ASCs showed a lower telomerase activity compared to the positive control (cancer cells) included in the kit which is consistent with previous reports^43^. Assuming that telomerase activity is associated with longer life span, self-renewal, and stemness, ASCs may have superior potential in regenerative therapies.

We have applied a novel technique to measure the electrokinetic properties of both ASCs and BM-MSCs in an electric field. In addition to biological differences, a marked variation in cell electrical properties was reported in the traveling wave speed and the rotation speed of ASCs and BM-MSCs. To our knowledge, this is the first report to compare the electrical differences between ASCs and BM-MSCs. This novel technique provides a valuable tool to measure different physical characteristics of cells, and may subsequently relate them to function. The difference in electrical properties between ASCs and BM-MSCs may correspond to differences in cell membrane structure, mitochondria or electrically charged biological components^44^. Electrons and other negatively charged anions such as phosphate ions account for negative charge, while protons, positively charged potassium, sodium, potassium, calcium and magnesium ions account for positive charges^45^. These charges account for cell conductivity and may correspond to specific biological differences.

These physical and functional differences between BM-MSCs and ASCs may be attributed to different cell origin, different cell populations or differences in cell structure. Since MSCs are composed of different types of precursor cells rather than having a pure cell population^46^, clonal analysis can provide more valuable insight into these differences.

Stem cell based therapies are for the most part experimental, with few published clinical trials. In our recent meta-analysis^47^, we have shown that a literature search on stem cell therapy in Diabetes Mellitus resulted on more than 4000 papers, however, only 22 clinical trials were reported. This gap between the benchtop and the clinic is due- in part- to the lack of starting with a well-defined, superior population of stem cell. Choosing the best stem cell type for clinical applications should not be based just on their differentiation capacity, but on factors related to the required effect. In many cases, immune reconstitution, replacement of diseased cells with healthy ones, and resistance of stem cells to senescence are all highly desirable. In cases of ischemia, the resistance to stress-induced injury, and vascular genesis abilities of ASCs are highly desirable. Taking into account the advantages of ASCs discussed above, ASCs may represent a highly promising resource for stem cell based therapies and an alternative to BM-MSCs.

## Material and Methods

### Animals

Male Sprague-Dawley rats (6–8 weeks old) were obtained from the National Research Center and were maintained at the Animal Research facility at Zewail City of Science and Technology following a protocol approved by the Animal Ethical Care and Use Committee in accordance with the National Institutes of Health Guide for the Care and Use of Laboratory Animals. The rats were kept at a temperature of 23 °C in a 12-hour light/dark cycle, and fed *ad libitum*.

### Isolation and characterization of MSCs

Rats were sacrificed by cervical dislocation and the tibia and femur were dissected. Low-glucose DMEM supplemented with 10% fetal bovine serum and 1% Pen-Strep (Life Technologies, USA) was injected into the dissected bone to collect the marrow cells. Cell suspension was passed through a 70 μm cell strainer (Greiner, Germany) to remove the debris. The cells were centrifuged at 200 g for 10 minutes and the supernatant removed by aspiration. The cells were re-suspended in complete culture media and seeded in 25 cm^2^ culture plates. Adherent cells were detached with 0.25% trypsin (Sigma-Aldrich, USA), centrifuged, re-suspended and plated in culture plates at 37 °C with 5% CO_2_. For most experiments, we used the 3rd passage of BM-MSCs.

To isolate ASCs, the inguinal fat pads from the rats was minced, and digested for 1 hour at 37 °C using collagenase type I (Gibco, USA). The reaction was stopped with the addition of supplemented media, pelleted via centrifugation, and subjected to erythrocyte lysis in accordance with the instructions of the manufacturer (Sigma-Aldrich, USA). Cells were cultured in 37 °C, 5% CO_2_ in DMEM with 10% FBS and 1% penicillin/streptomycin (Life Technologies, USA) containing 1 g/L of glucose. When the adherent cells reached confluence, they were detached with 0.25% trypsin (Sigma-Aldrich, USA) and re-suspended in supplemented media. For most experiments, we used the 3rd passage of ASCs.

The differentiation capacity of MSCs into adipocytes and osteoblasts was evaluated. Briefly, BM-MSCs and ASCs were seeded in standard six-well tissue culture plates (1.5 × 10^5^ cells per well), and adipogenic differentiation medium—consisting of DMEM (1 g/L glucose), 10% fetal bovine serum, 1% penicillin/streptomycin, 10 μg/mL insulin, 1 μM dexamethasone, 0.5 mM methylxanthine, and 200 μM indomethacin—were added after cell attachment. Oil red O staining was performed after 7 days of incubation. In addition, osteogenic differentiation medium was added to other cells, which consisted of DMEM (1 g/L glucose) supplemented with 10% FBS, 1% penicillin/streptomycin, 100 μg/mL ascorbic acid, and 10 mM β-glycerophosphate. Photometric quantification of Alizarin red stain was performed after 14 days to assay extracellular mineralization as previously described^48^.

### Immunophenotypic characterization of BM-MSCs and ASCs

For the flow cytometry analysis, cells were incubated in a blocking solution (PBS containing 1% BSA and 1% FBS) for 10 minutes. After centrifugation, cells were re-suspended in the blocking solution and were stained with the following antibodies for 30 minutes: FITC anti-CD90 mAb, PE anti-CD29 mAb and APC anti-CD45 mAb. Cells were acquired on a FACSCalibur (Becton Dickinson, USA) following standard procedures and analyzed using CellQuest Pro Software (Becton Dickinson, USA).

### Induction of hind limb ischemia and cellular therapy

Ischemia was induced surgically in 6–8 weeks old Male Sprague-Dawley rats as previously described^49^ with slight modifications. Briefly, rats were fasted for 12 h before surgery, and intraperitoneally anesthetized using ketamine (100 mg/kg) and xylazine (5 mg/kg). Under aseptic conditions, a longitudinal incision was made in the right hind limb 2–3 cm from the femoral artery. The proximal portion of the femoral artery including the superficial and the deep branches were ligated twice with 5–0 sterile silk suture, and the overlying skin was closed. Sham operated animals underwent left femoral artery exposure without ligation to serve as non-ischemic controls.

At 24 h post-induction of hind limb ischemia, the rats were randomly divided into four groups (n = 3 per group): non-ischemic animals (sham), ischemic untreated animals (control), and ischemic animals treated with BM-MSCs and ischemic animals treated with ASCs. After 7 days of ischemia induction, 2 × 10^6^ cells in 0.5 mL PBS were injected at intramuscularly three different sites of the ischemic leg using a 21 G needle. MSCs with passage numbers between 3 and 5 were used in these experiments. Animals in both the sham and control group received an injection of 0.5 mL PBS alone into the ischemic limb. All studies were repeated twice.

### Histological and Immunohistochemistry analysis

After 3 weeks of MSC therapy, the animals were euthanized and the muscles removed and washed with PBS. The ischemic muscles were fixed in 4% paraformaldehyde for 24 hours, dehydrated and embedded in paraffin. Sections of 5 μm were obtained and used for staining with hematoxylin and eosin (H & E) to determine muscle regeneration and infiltrated cells. Paraffin sections (5 μm) taken from the ischemic and non-ischemic limbs were deparaffinized, and subjected to antigen retrieval followed by immunohistochemistry using anti-CD31, anti-CD34, anti-VEGF, anti-smooth muscle actin (SMA) and anti-matrix metalloproteinase 9 (MMP9) monoclonal antibodies. The total number of CD31, CD34 and SMA positive cells in 4 photographic fields of each slide were counted and considered as a quantification of the expression levels of each marker. The extracellular staining intensity of VEGF and MMP9 was scored semi—quantitatively as previously described with coring levels (1 = weak staining; 2 = moderate staining; and 3 = strong staining)^50^,^51^,^52^,^53^. All antibodies were purchased from Santa Cruz Biotechnology Inc., Germany.

### Oxidative stress-induced senescence and β-galactosidase (SA-β-Gal) assay

Briefly, both BM-MSCs and ASCs were cultured in 6-well plates and H_2_O_2_ treatment was carried out 24 hours after seeding in media containing 600 μM H_2_O_2_ for 2 hours^54^. After 4 days, a second dose of 600 μM H_2_O_2_ was added to the cells for 2 more hours. For assessment of SA-β-gal activity, the cells were fixed with 4% paraformaldehyde for 15 min, washed twice with PBS, and stained with a SA-β-gal detection kit (BioVision Inc., USA) overnight at 37 °C and according to the manufacturer’s instruction. Cells were examined under a Leica DMi8 phase contrast inverted microscope (Leica Microsystems, Germany). Cellular death after H_2_O_2_ treatment was measured by flow cytometry using Annexin-V-FITC and PI apoptosis detection kit (Miltenyi Biotec Inc., USA) according to the manufacturer’s protocol.

### MTT assay

The proliferation of BM-MSCs and ASCs after H_2_O_2_ treatment was assessed using the 3-(4,5-dimethylthiazol-2-yl)-2,5-diphenyltetrazolium bromide (MTT) assay (Life Technologies, USA) as previously described. Briefly, after H_2_O_2_ treatment, MTT (5 mg/ml) was added to each well of BM-MSCs and ASCs and incubated in a humidified 5% CO_2_ incubator at 37 °C for 3 hours. The formazan salts were dissolved with DMSO for 15 minutes and the optical density was measured at 570 nm with reference to 630 nm by using a FLUOstar Omega-microplate reader (BMG Labtech, NC).

### Hypoxia conditioning and measurement of cell survival and apoptosis

BM-MSCs and ASCs were plated in 10% FBS supplemented media on 6-well plates (1 × 10^6^ cells per well) and cultured in hypoxic conditions (1% O_2_/5% CO_2_/94% N_2_) for 24 hours at 37 °C in a sealed humidified chamber. Hypoxia conditions were automatically adjusted by an electronic oxygen controller, then phase contrast images of BM-MSCs and ASCs were taken using a Leica DMi8 inverted microscope (Leica Microsystems, Germany) and percentage of apoptotic cells was detected by flow cytometry using Annexin-V-FITC and PI apoptosis detection kit (Miltenyi Biotec Inc., USA) according to the manufacturer’s protocol. Cells were analyzed using a FACSCalibur (Becton Dickinson, USA) and CellQuest Pro Software (Becton Dickinson, USA).

### *In vitro* angiogenesis tube formation assay

Geltrex^®^ LDEV-Free Reduced Growth Factor Basement Membrane Matrix (Invitrogen, USA) was thawed at 4 °C overnight before use. Geltrex^®^ matrix was added in 24-well plate (100 μl/well) and incubated at 37 °C for 30 min to allow solidification. Both BM-MSCs and ASCs at passage 4 were seeded on the coated wells at the density of 1.5 × 10^6^ in 250 μl of large vessel endothelial-supplemented Medium 200 (Gibco, USA) and incubated overnight at 37 °C in a humidified atmosphere of 5% CO_2_. At 16 hours post-seeding, cells were stained with 2 μg/mL of Calcein, AM (Molecular Probes, USA), incubated for 30 minutes at 37 °C, 5% CO_2_, and then imaged at 4x magnification using a Leica DMi8 inverted fluorescent microscope (Leica Microsystems, Germany). The total number of branching points in 4 photographic fields of each well were considered indicative of the complexity of the formed capillary network. All experimental conditions were tested in triplicates.

### Telomerase activity measurement

To test telomerase activity, BM-MSCs and ASCs were harvested, counted and lysed at 4 °C in CHAPS lysis buffer for 30 minutes, and then centrifuged at 12,000 × g for 20 minutes at 4 °C. The clear cell lysates were assayed for telomerase activity using a TRAPeze^®^ XL Telomerase Detection (fluorescence based detection) kit (Millipore Corp, USA; Cat #S7707) according to the manufacturer’s instructions. Briefly, for the telomerase assay, reaction mixtures containing 1 μg of protein from extracts of BM-MSCs and ASCs were assayed. Dilution series of TSR8 control template was prepared in CHAPS lysis buffer to serve as a standard curve. As negative controls, extracts were heat-treated for 10 min at 85 °C, and one reaction mixture was run without any extract. A positive control is included in the kit. Each reaction was performed in triplicates and samples were subjected to the following cycling parameters using a thermocycler: 30 min at 30 °C (extension of telomerase substrate); 94 °C/30 seconds, 59 °C/30 seconds, 72 °C/1 minute for 36 cycles followed by a 72 °C/3-minute extension step and then at 55 °C/25 minutes, concluding with a 4 °C incubation. The PCR reaction product was fluorometrically detected using a fluorescent microplate reader (FLUOstar Omega microplate reader; BMG Labtech, NC). The quantity (amoles) of extended telomerase substrate produced in each well was determined from a linear plot of the log_10_ of the quantities (amoles) of TSR8 control template standards versus the Ct values for their wells. The mean value of these quantities for the three replicate wells for each sample was calculated.

### Real-time qPCR

RNA was extracted from BM-MSCs and ASCs using the PureLink^®^ RNA Mini Kit (Life Technologies, USA) according to the manufacturer’s instructions and treated with DNAse I (Sigma-Aldrich). The cDNA was synthesized by using the High Capacity Reverse Transcription cDNA kit (Life Technologies, USA) and quantitative Real-Time PCR assay was performed using SYBR^®^ Premix Ex Taq™ II (Takara, Japan) in the QuantStudio™ 12 K Flex Real-Time PCR System (Applied Biosystems, USA). The sequence of primers used are indicated in Supplementary Table S1. The relative gene expression was calculated by 2^−ΔΔCT^ method and the β-actin gene was used to normalize the data. Each reaction was performed in triplicates, and each experiment was performed twice.

### Microfluidic platform Setup

In order to make the electrode sets work together, a good signal generating and control system is required. A function generator setup from an electrokinetic based microfluidic platform (Supplemental Fig. 2) was used to produce a square wave signal and its 90° phase shifted signal. The output signals from the function generator were fed into a unity gain inverting amplifier circuit (using current conveyer, AD844), which produced two identical 90-degree phase shifted signals. Thus, four identical signals with 0°, 90°, 180° and 270° phase shift, respectively, were produced. Dip switches were utilized to control and activate certain set of electrode configuration. Supplemental Fig. 2B shows the block diagram of the system setup. Experimental phenomena were monitored using an Olympus microscope with an attached video camera, connected to a laptop. Simultaneously, an oscilloscope was connected in order to confirm the continued operation of the stimulus signal.

### Microelectrode array used to differentiate between ASCs and BM-MSCs

The platform included an electrokinetic module and was implemented using printed circuit board (PCB) technology where cells were placed on a microelectrode array (Supplemental Fig. 2C). A concentric ring electrode structure for dielectrophoresis (DEP) force has been used^55^,^56^,^57^,^58^. The efficacy of this design is proven and is compared to a similar design previously described^59^. The proposed PCB platform has four sets of electrodes with similar geometric configuration. All of these were aggregated onto a single PCB using PCB technology. Each single electrode is 150 μm wide and is spaced at 150 μm intervals. Each set consists of 20 individual electrodes. On energizing the platform with sequential signals of relative phase, as indicated in Supplemental Fig. 2B, a Travelling Wave field propagates either radially from the center to the periphery of the device or from right to left parallel to the X direction within the central area of the platform. For rotation, switches are adjusted to generate an anti-clockwise rotating electric field in the left or right central region respectively.

### Electrokinetic Module to determine different features of ASCs and BM-MSCs

The electrokinetic module (Supplemental Fig. 2C) was developed to determine the following functions: (1) Travelling wave conveyance to facilitate both the transfer of both ASCs, or BM-MSCs from the periphery of the chamber toward the central region, for subsequent levitation and/or electrorotation, and then removing these cells from this central region; (2) Cell Rotation; and (3) Cell Levitation. Each function (i.e., traveling, rotation or levitation) is fully controlled by the control unit.

### Statistical analysis

All results are presented as mean ± SD. Comparisons between groups were analyzed by use of Student’s *t* test. Probability values of P < 0.05 were considered statistically significant.

## Additional Information

**How to cite this article**: El-Badawy, A. *et al*. Adipose Stem Cells Display Higher Regenerative Capacities and More Adaptable Electro-Kinetic Properties Compared to Bone Marrow-Derived Mesenchymal Stromal Cells. *Sci. Rep.* **6**, 37801; doi: 10.1038/srep37801 (2016).

**Publisher's note:** Springer Nature remains neutral with regard to jurisdictional claims in published maps and institutional affiliations.

## Supplementary Material

Supplementary Figures and Table

Supplementary Video 1

Supplementary Video 2
