# Supplementary material for: Adipose Stem Cells Display Higher Regenerative Capacities and More Adaptable Electro-Kinetic Properties Compared to Bone Marrow-Derived Mesenchymal Stromal Cells
Source: Sci Rep. 2016 Nov 24;6:37801. doi: 10.1038/srep37801 (PMC5121630; doi:10.1038/srep37801)
Supplement: Supplementary Figures and Table [file srep37801-s1.pdf]

Adipose Stem Cells Display Higher Regenerative Capacities and More Adaptable Electro-Kinetic Properties Compared to Bone Marrow-Derived Mesenchymal Stromal Cells

Ahmed El-Badawy[1], Marwa Amer[1], Reda Abdelbaset[2,3], Sameh Elmasry[2,3], Marwan Abo-Elela[2], Yehya H. Ghallab[2,3], Hamdy Abdelhamid[2], Yehea Ismail[2] and Nagwa El-Badri[1]*

1. Center of Excellence for Stem Cells and Regenerative Medicine (CESC), Zewail City of Science and Technology, Egypt
2. Center of Nanoelectronics and Devices (CND), Zewail City of Science and Technology/ American University in Cairo, Cairo, Egypt
3. Department of Biomedical Engineering, Helwan University, Cairo, Egypt

*** Correspondence:** Prof. Nagwa El-Badri, M.D., Ph.D.
Director, Center of Excellence for Stem cells and Regenerative Medicine (CESC),
Zewail City of Science and Technology, Sheikh Zayed District, 12588, 6th of October City, Giza, Egypt, Tel: +2 02 385 40 401, Email: nelbadri@zewailcity.edu.eg

Supplementary Figure 1

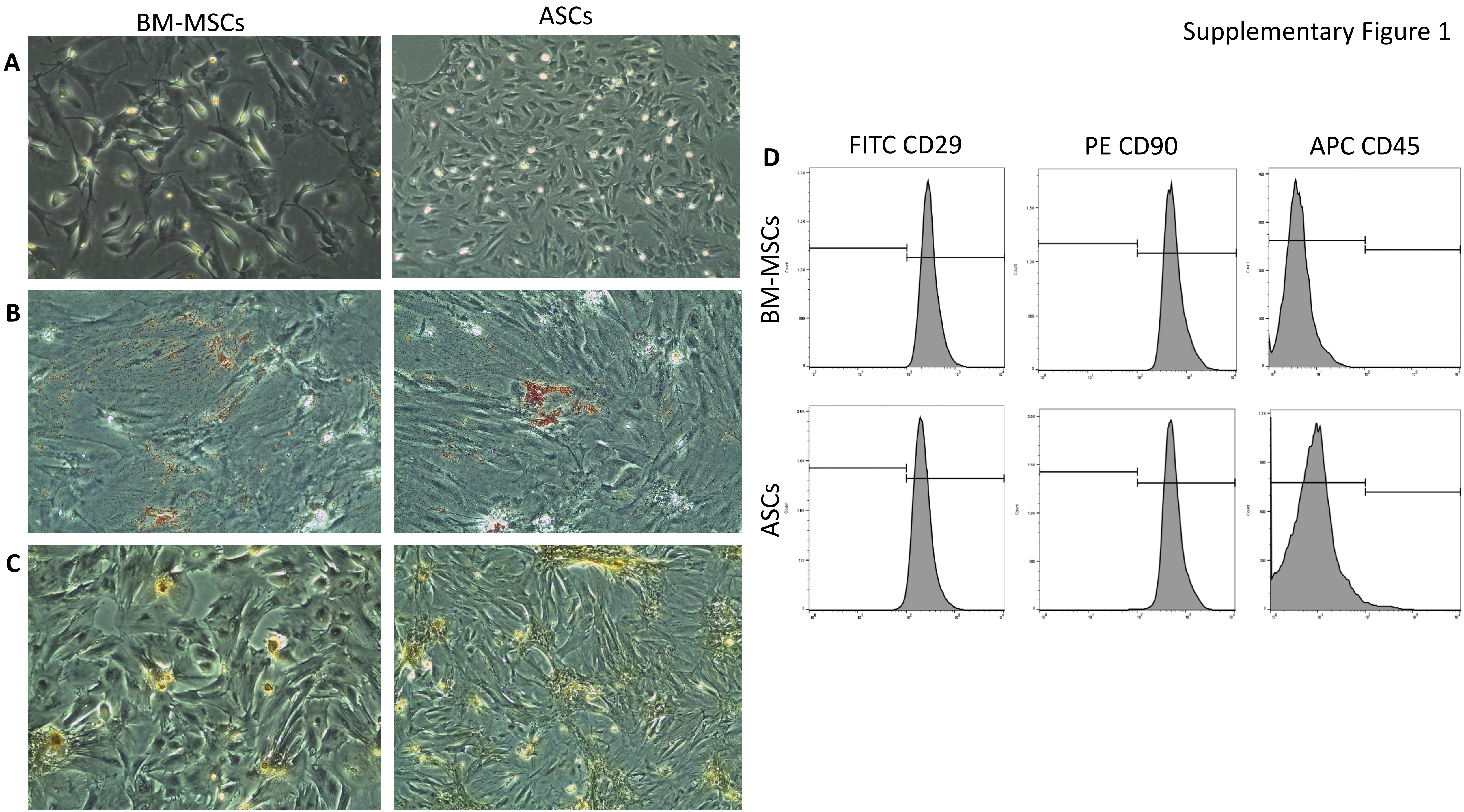

CD31

Supplementary Figure 2

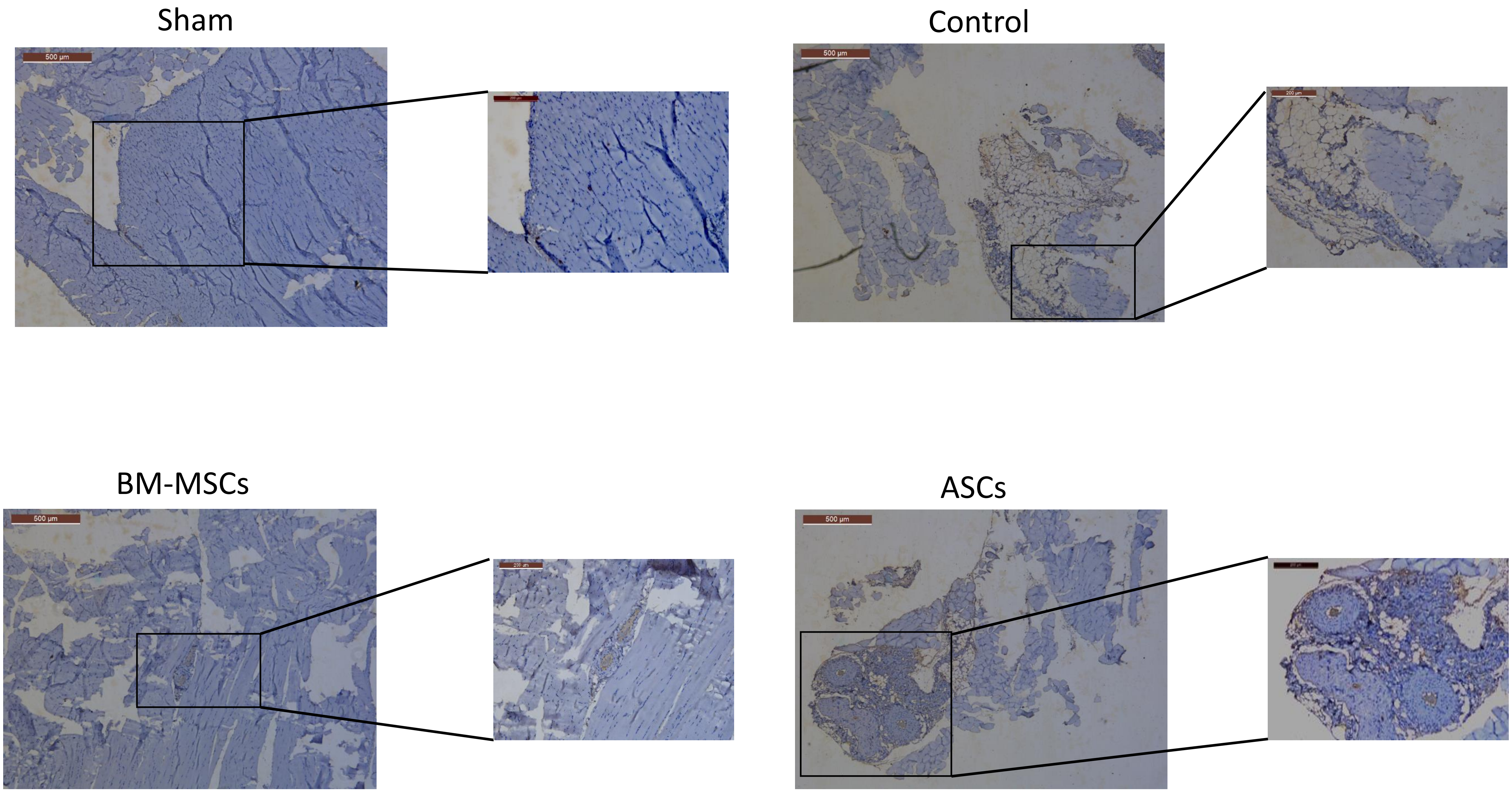

CD34

Supplementary Figure 3

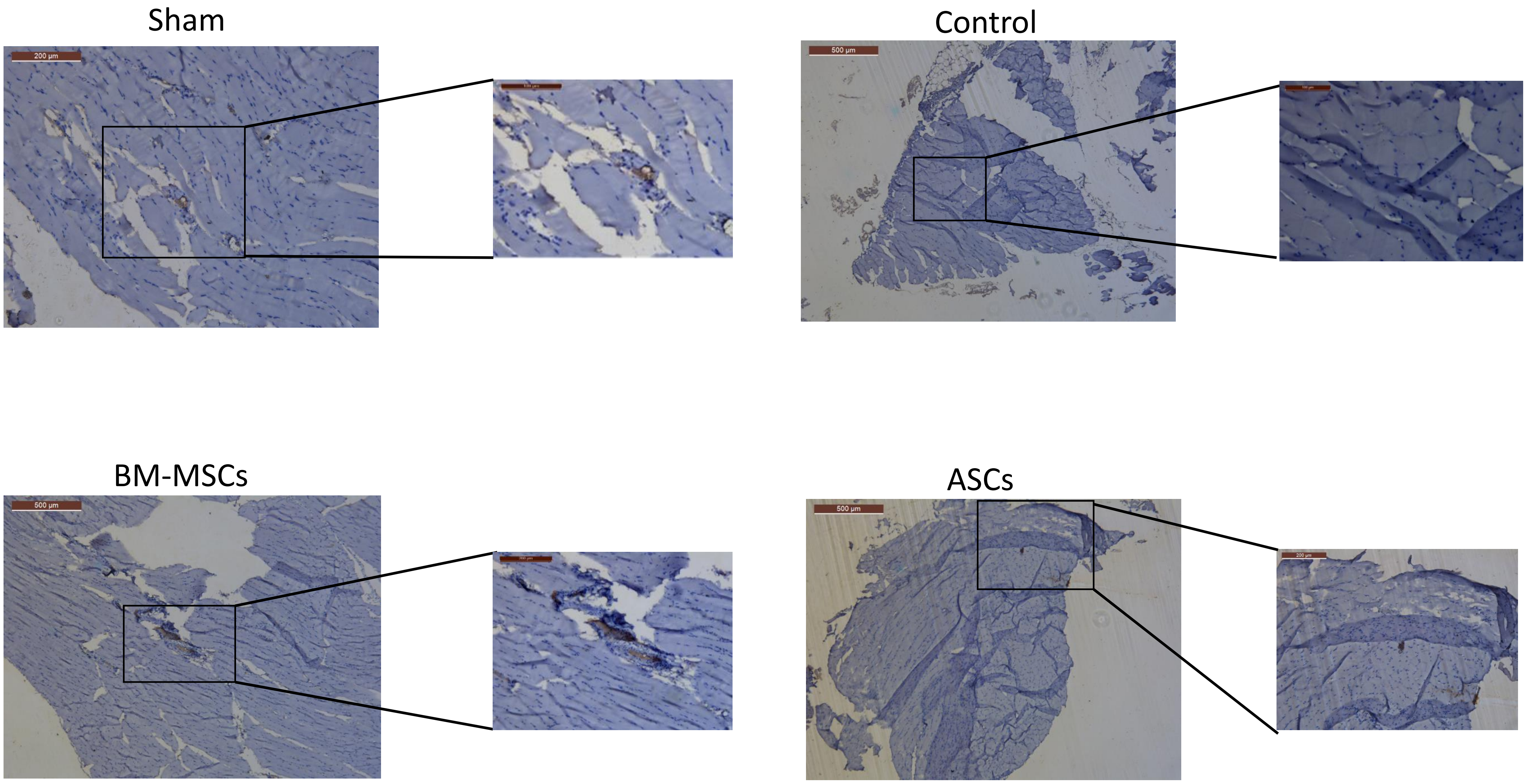

VEGF

Supplementary Figure 4

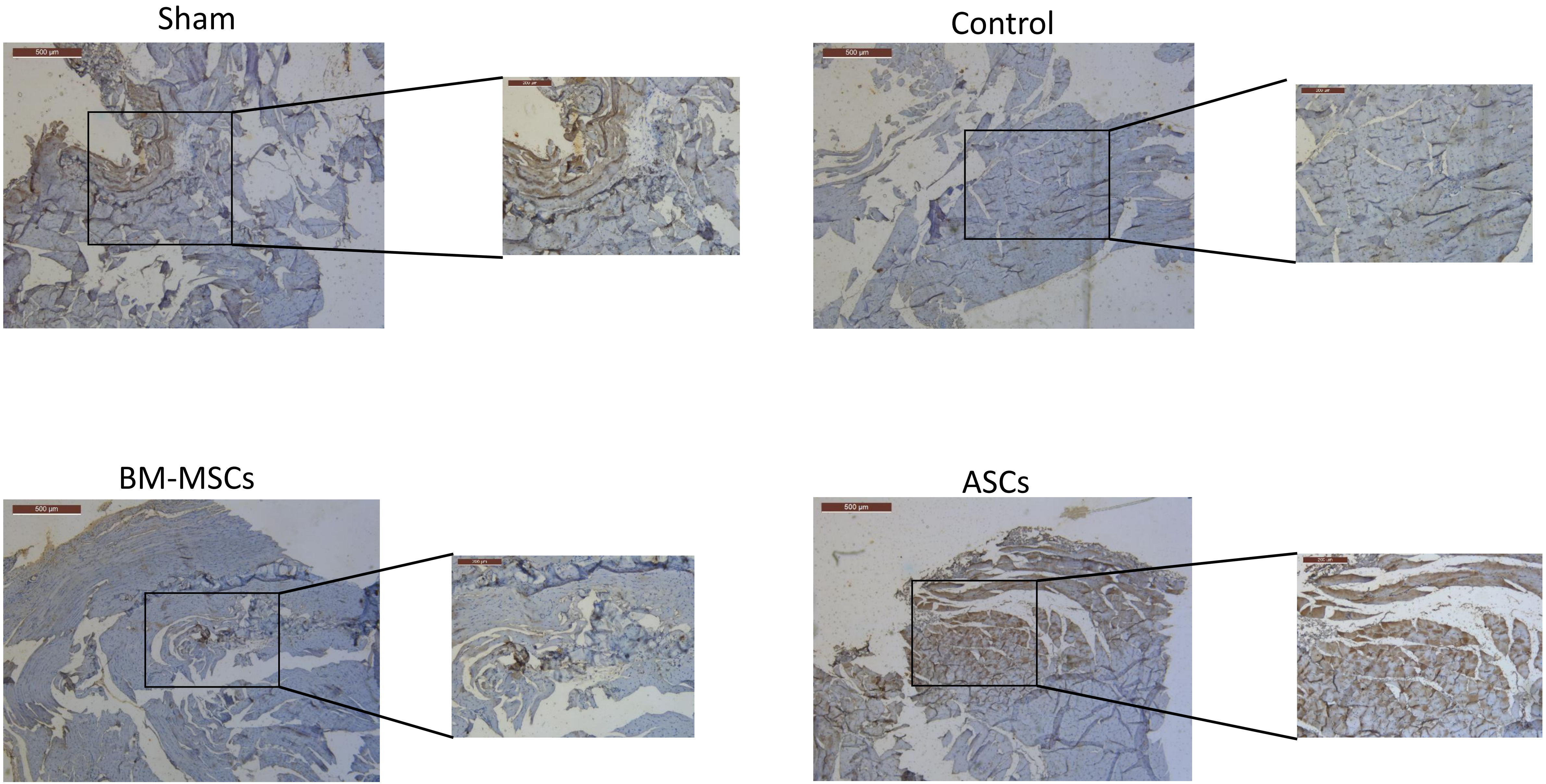

αSMA

Supplementary Figure 5

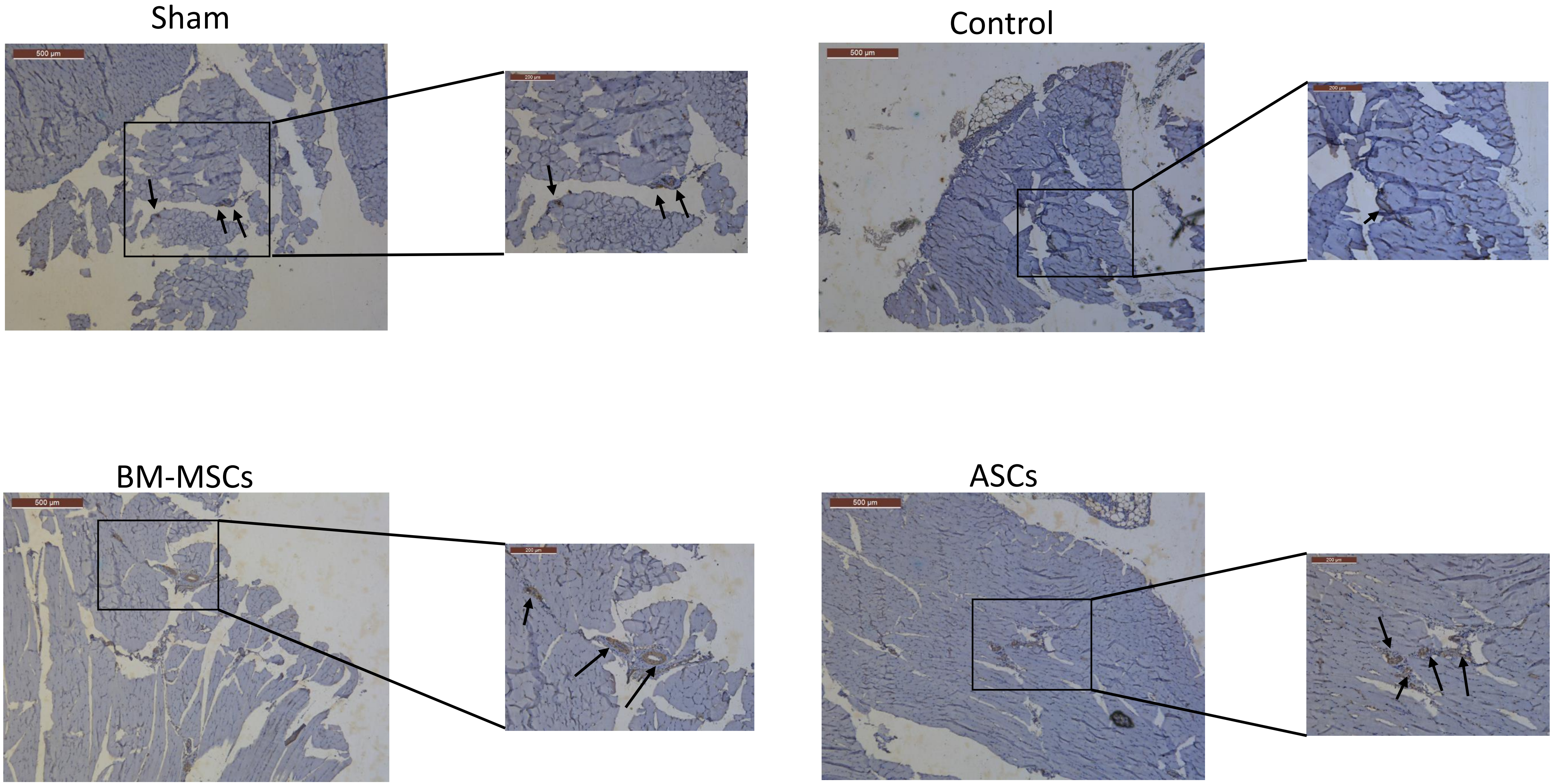

MMP9

Supplementary Figure 6

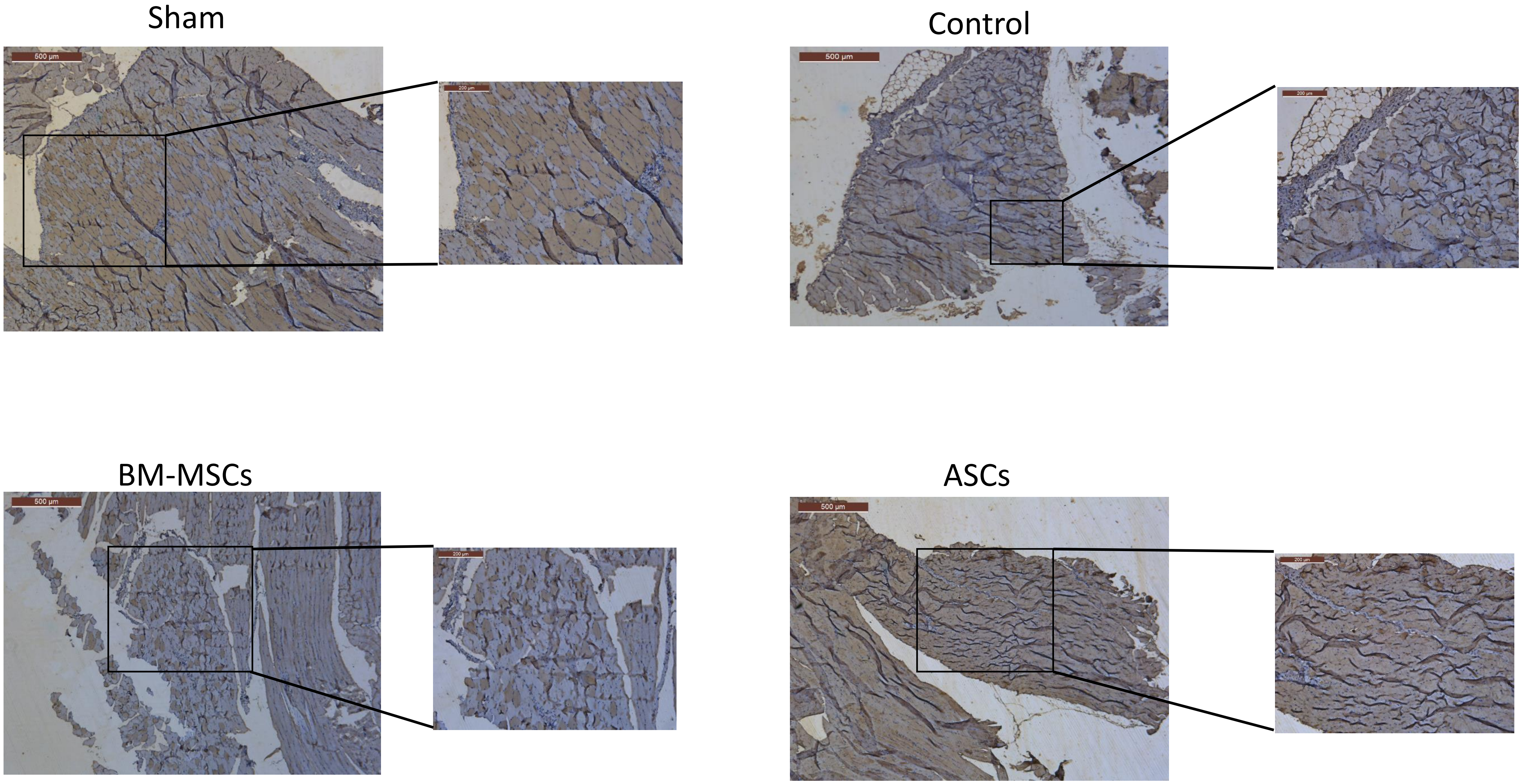

Supplementary Figure 7

A

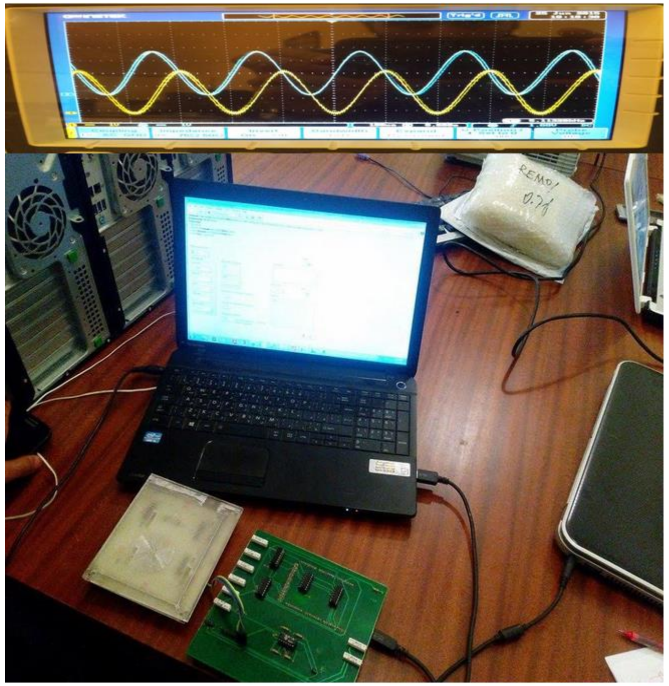

B

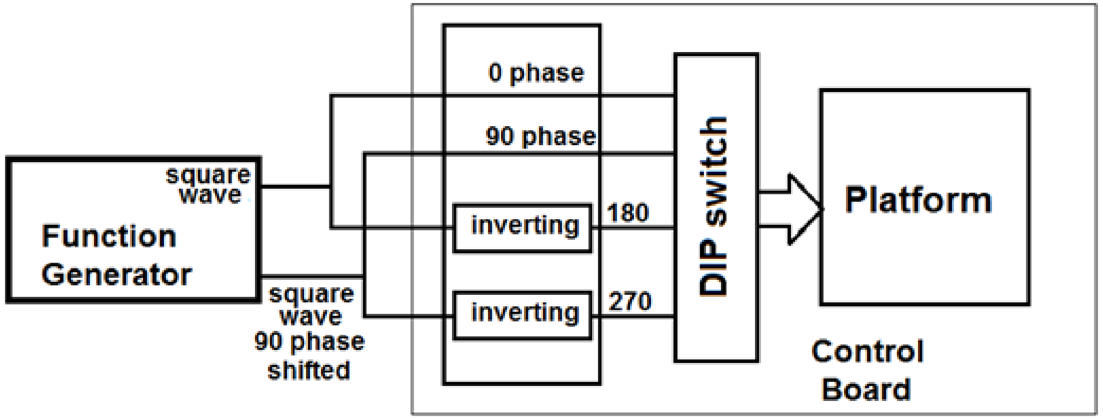

C

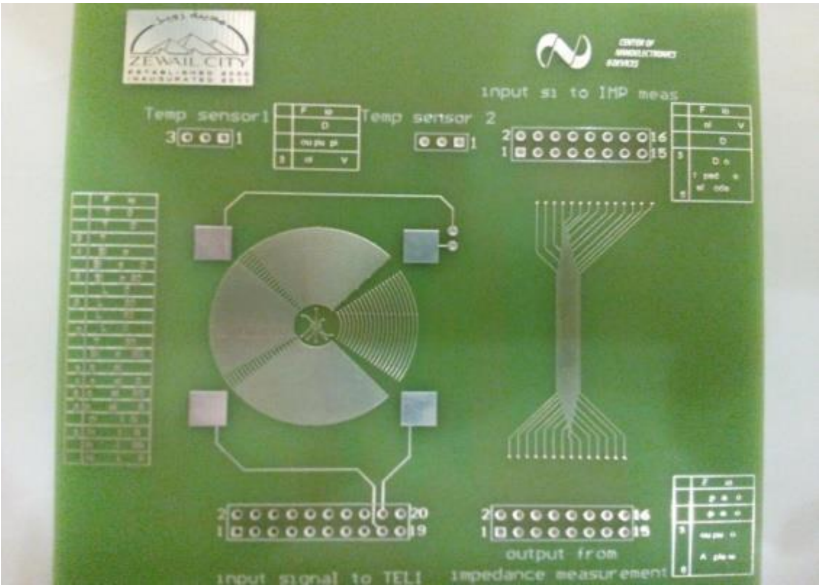

Supplementary Figures Legend:

Figure 1: Phase contrast images of BM-MSCs and ASCs showing elongated spindle-shaped cells morphology (A). Phase contrast images of BM-MSCs and ASCs differentiated into adipocytes as indicated by oil red O staining (B) and into osteocytes as indicated by alizarin red staining (C). (D) Flow cytometry characterization of BM-MSCs and ASCs showing a weak expression of the leukocyte marker, CD45, and positive expression of stem cell-specific markers, CD29 and CD90. Scale bar, 100 µm. Magnification 10x

Figure 2: Representative histological analysis of original and magnified images of hind limb muscles stained for CD31

Figure 3: Representative histological analysis of original and magnified images of hind limb muscles stained for CD34

Figure 4: Representative histological analysis of original and magnified images of hind limb muscles stained for VEGF

Figure 5: Representative histological analysis of original and magnified images of hind limb muscles stained for αSMA

Figure 6: Representative histological analysis of original and magnified images of hind limb muscles stained for MMP9

Figure 7: (A) Complete Microfluidic Platform which includes the PCB platform, microfluidic chamber and the laptop (B) Block diagram of the system setup which includes the switches and the generator interface (C) PCB platform used to extract electrokinetic properties of ASCs and BM-MSCs

Tables:

Supplementary Table S1: Sequence of Primers used

Gene name	Primer Sequence
Beta actin- Forward	ATCATGTTTGAGACCTTCAACACC
Beta actin- Reverse	TAGCTCTTCTCCAGGGAGG
Oct4- Forward	TCAGGTTGGACTGGGCCTAGT
Oct4 -Reverse	GGAGGTTCCCTCTGAGTTGCTT
VEGF- Forward	GCACTGGACCCTGGCTTTACT
VEGF -Reverse	ATGGGACTTCTGCTCTCCTTCTG
TGF beta1- Forward	ATACGCCTGAGTGGCTGTCT
TGF beta1- Reverse	TCTCTGTGGAGCTGAAGCAA
FGF-2- Forward	CTTGCTATGAAGGAAGATGGA
FGF-2 Reverse	TGCCCAGTTCGTTTCAGTG
MMPII- Forward	TTGCAGGAGACAAGTTCTGGAG
MMPII- Reverse	CGATGAGCTTAGGGAAAC

Supplementary Videos Legend:

Online Supplementary Video 1: Traveling wave velocity of ASCs and at 8 MHz

Online Supplementary Video 2: Traveling wave velocity of BM-MSCs and at 8 MHz
